# Supplementary material for: A Unique Anti-Cancer 3-Styrylchromone Suppresses Inflammatory Response via HMGB1-RAGE Signaling
Source: Medicines (Basel). 2021 Mar 24;8(4):17. doi: 10.3390/medicines8040017 (PMC8064355; doi:10.3390/medicines8040017)
Supplement: Supplementary file 1 [file medicines-08-00017-s001.pdf]

Supplementary Materials: A Unique Anti-Cancer 3-Styrylchromone Suppresses Inflammatory Response via HMGB1-RAGE Signaling

Hideaki Abe, Miwa Okazawa, Takahiro Oyama, Hiroaki Yamazaki, Atsushi Yoshimori, Takanori Kamiya, Mitsutoshi Tsukimoto, Koichi Takao, Yoshiaki Sugita, Hiroshi Sakagami, Takehiko Abe and Sei-ichi Tanuma

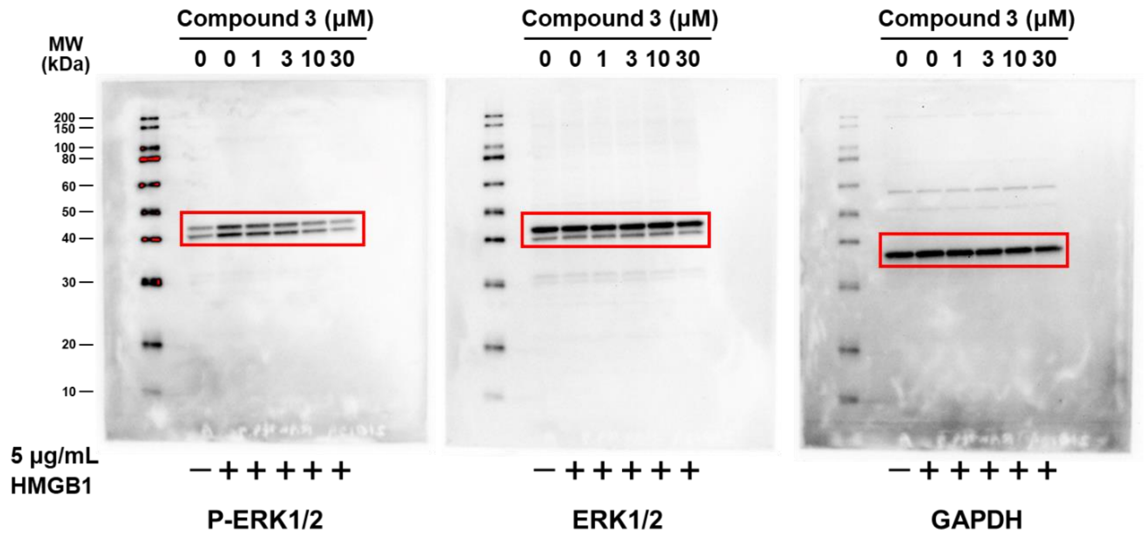

Figure S1. Original scans for the immunoblots in Figure 4. Western blotting of samples was performed as described in the Materials and Methods.
